# Chemical named entities recognition: a review on approaches and applications

**DOI:** 10.1186/1758-2946-6-17

**Published:** 2014-04-28

**Authors:** Safaa Eltyeb, Naomie Salim

**Affiliations:** 1 Faculty of Computing, Universiti Teknologi Malaysia, Johor, Malaysia; 2 College of Computer Science and Information Technology, Sudan University of Science and Technology, Khartoum, Sudan

**Keywords:** Chemical entities, Information extraction, Chemical names

## Abstract

The rapid increase in the flow rate of published digital information in all disciplines has resulted in a pressing need for techniques that can simplify the use of this information. The chemistry literature is very rich with information about chemical entities. Extracting molecules and their related properties and activities from the scientific literature to “text mine” these extracted data and determine contextual relationships helps research scientists, particularly those in drug development. One of the most important challenges in chemical text mining is the recognition of chemical entities mentioned in the texts. In this review, the authors briefly introduce the fundamental concepts of chemical literature mining, the textual contents of chemical documents, and the methods of naming chemicals in documents. We sketch out dictionary-based, rule-based and machine learning, as well as hybrid chemical named entity recognition approaches with their applied solutions. We end with an outlook on the pros and cons of these approaches and the types of chemical entities extracted.

## Introduction

Scientific results are most commonly presented in the form of scientific articles, industry reports, or thesis documents. Normally these documents are written in natural languages mixed with domain-exclusive terminologies added to numerical data. Thus, they are rich with unstructured data that cannot be understood by a machine. As a result, reusing these data is not an easy matter. Manual information extraction from the literature by humans has become a business managed by information providers. However, manual information extraction is costly. Obtaining the extracted information after publication is often time consuming and fallible [1].

Specific information on newly discovered compounds is often difficult to find in chemical databases. For example, drug research requires the knowledge of new molecules for developing new drugs. Researchers may also want to search for potential lead compounds or determine the function of the compound. Obtaining previous knowledge on chemicals, such as biological properties or toxic effects, can help in many aspects of drug development processes. The entities extracted can be linked to their properties or co-occurrence with other entities, which can allow us to identify new knowledge between them. Finding mentions of chemical compounds in the texts is useful for many reasons, including mapping entities to corresponding structures to find relationships between chemicals. Chemists can then search for similar structures or substructures, and the knowledge in the text can be combined with the knowledge from chemical databases. The annotation of entities enables a search engine to return documents that contain elements of this entity class, such as their activities, which can be helpful to find other relationships, such as adverse reactions or diseases [2]. Thus, the analysis of information found in the texts seems unavoidable because text-mining tools can greatly augment, improve and facilitate this process of information extraction. However, the variety of naming standards for chemical entities makes this task extremely complex and time consuming. Hence, this task should be supported by computational tools.

## Review

This paper unites the types of chemical entities with computerised methods for extraction to help practitioners entering into this area. Thus, different types of chemical entities supported by examples are organised according to the taxonomy derived from the literature. The methods of recognising the names of chemical entities are then surveyed accompanied by relevant references and summaries for all solutions starting from 2000, and they are then correlated with the types of chemical entities extracted.

The rest of this paper is organised as follows. Section Chemical literature mining is a background section that gives an overview of chemical literature mining. Section Evaluation introduces the corpora and evaluation methods. Section Chemical Named Entity Recognition (NER) approaches presents the methods of NER that are applied in the chemical entity recognition. Section Discussion presents an outlook on the applied methods and extracted entities. Section Conclusion concludes the paper.

## Chemical literature mining

Although chemical information mining was mentioned in chemistry before biology [3], text mining is not widespread and fewer tools have been developed [1]. An example of the extracted information from the biology literature is information on genes and proteins and their functional relationships. Reasons for mining chemical entities from the literature include the following:

• To identify unique chemical entities.

• To index the bibliographic chemical databases [4].

• To link between chemical structures and biological processes [5].

### Sources of chemical information

Some, but not all, chemical information is freely available. Many types of chemical databases are available:

➢ Chemical structure databases.

➢ Chemical literature databases.

➢ Nuclear Magnetic Resonance (NMR) spectra databases.

➢ Crystallographic databases.

➢ Reactions databases.

Chemical literature databases associate structures or other chemical information with the relevant documents. Many free and commercial databases cover chemistry literature and structure. They vary in terms of time period of coverage, frequency of update, publication type (e.g., journals, books, chapters, theses, and technical reports) and the type of search provided (e.g., search by chemical names, trade names, molecular structure or keywords etc.). These databases include the following:

➢ PubMed® ^a^and PubMed Central ^b^(PMC®), which cover the biomedical literature from MEDLINE® ^c^and life sciences journals and online books. They are managed by the National Center for Biotechnology Information (NCBI), a component of the U.S National Library of Medicine (NLM). PMC carries the full text of the paper, whereas PubMed includes only the citations and abstracts of papers.

➢ PubChem^d^, which is a database of chemical molecules. The system is maintained by NCBI. It contains substance descriptions and small molecules as well as links to the PubMed scientific literature.

➢ ChemSpider^e^, a free database providing access to structures, properties, and their related information. It enables searches with text and structures and provides important data, such as literature references, physical properties and chemical suppliers.

➢ SciFinder^f^, which is used to access information in selected Chemical Abstracts Service (CAS^g^) databases. It offers a variety of searches: CAS Registry Number, author name, research topic, or chemical structure/substructure.

Normally, chemical documents, such as theses, lab books, industry reports, journal articles and patents found in text (e.g. text, rich text format, and word documents), are embedded with figures and/or tables [5].

### The textual contents of chemical documents

**Figure 1:**
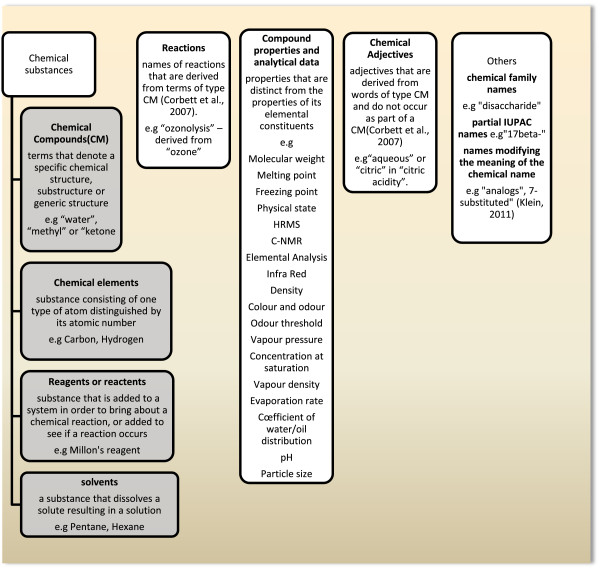
Example of classes of chemical entities-bolded-extracted by different systems from the chemical literature.

A manual analysis of 20 papers from Organic & Bimolecular Chemistry [6] reported a need for chemistry-specific lexicons for various concepts, such as actions, quantities, substance, states, procedures, etc. Other examples of the contents in chemical patents are compounds, reagents and solvents [7]. Figure 1 shows the classes of named chemical entities mined by different systems as described by examples.

### Methods for expressing chemical structural information in documents

The main source of difficulty in mining chemical structural information from literature is the lack of a standardised naming convention to represent the chemical structural information. Different expressing methods and naming groups have been used in which chemical terms in documents can be assigned to structures as described in Table 1 with examples.

**Table 1:** **Description and examples of the expressing methods of chemical structural information (**[5,8]**and**http://en.wikipedia.org**)**

**Expressing method**	**Description**	**Example**
1. Systematic names	reflect the information of the chemical structure. International Union of Pure and Applied Chemistry (IUPAC^h^)	‘3-(3,4-dihydroxyphenyl)prop-2-enoic acid’
2. Trivial names	they do not reflect the structure of the chemical substance.	‘caffeic acid’ utilized for ‘3-(3,4-dihydroxyphenyl)prop-2-enoic acid’.
3. Semi systematic names	at least one part is used in the systematic sense, IUPAC-like, non-IUPAC names.	in‘N-benzoylglycine’ the part ‘benzoyl’ is systematic, whereas ‘glycine’ is the trivialname for ‘*_*-aminoacetic acid’
4. Common or generic names	names applied to a class of compounds	camphor, water and alcohol
5. Registered trademark/brand names	they identify the brand owner as the commercial source of products.	‘aspirin’
6. Company codes	a company code is to identify the compound within the company.	ZD5077 = ICI204636 = ZM204636
7. Acronyms and abbreviations	they are used to get short names.	DMS for dimethyl sulfate
8. Index and reference	numbers from Chemical Abstracts Service (CAS) registry numbers, Beilstein registry numbers, etc	CAS number of water is 7732-18-5
9. Anaphors	Compounds are named earlier in the text but co-referenced to a shorter name, called the anaphor, later in the text.	A compound number is anaphor where … bioactivity is found in compounds [1-7,9-11] listed in Additional file 1…’
10. Sum formula	Consists of the elements contributing to a compound and the number of their occurrences	‘*C*9*H*8*O*4’
11. Chemical structures	explicit and implicit structures	Markush structures, where R1 = CH3, COOH, etc…

The variety of methods used to represent chemical names and the variations of naming within one method itself (e.g. a systematic name can include multiple variations on how hyphens and dashes are located: 1,1- versus 11- versus 1–1-) [9] complicates chemical name recognition in text. Two-dimensional diagrams are the basic units used to represent chemical structures in chemistry. They are included in journal articles and patents as raster images. Optical Chemical Structure Recognition (OCSR) is used to extract the structural information from these images. Many systems implement OCSR, which can be found in the literature, such as in [10] and Park [11].

Thus, extracting information, such as extracting relationships between specific types of entities (as in [12-15], or inferring facts (such as in [16]) first requires that the mentions of the entities in the text be detected. This step is essential, and its success determines the success of other tasks of information extraction. In this paper we focus on information extraction in a natural language text that concerns the identification of instances of a specific class of entities in the text.

## Evaluation

Before presenting the Chemical Named Entity Recognition (CNER) approaches, Table 2 describes the available manually annotated text corpora for training and assessment of CNER tools according to the chemical entities focus, reference and source. However, due to the shortage of annotated corpus available for evaluation and training chemical NER systems, many developers of the systems generate their own corpus.

**Table 2:** Chemical text corpora for evaluating and training the NER applications

**Corpus**	**Class of named entities**	**Reference**	**Availability**
IUPAC training corpus	IUPAC names	[2]	http://www.scai.fraunhofer.de/chem-corpora.html
SCAI	All chemical names	[17]	http://www.scai.fraunhofer.de/chem-corpora.html
PubMed corpus	Compounds, reagents, chemical adjectives enzymes and prefix	[18]	Not available.
Sciborg corpus	All chemical names	[18]	Not available
GENIA corpus	Biological besides some chemical entities	[19]	http://www-tsujii.is.s.u-tokyo.ac.jp/GENIA
European Patent Office and the ChEB	All chemical names	[20]	http://chebi.cvs.sourceforge.net/viewvc/chebi/chapati/patentsGoldStandard
CHEMDNER Corpus	Chemical compounds and drugs	[21]	http://www.biocreative.org/tasks/biocreative-iv/chemdner/

To evaluate the performance of NER applications, the known information extraction measures are used, which are: (i) precision to measure the ability of a system to present only the relevant names; (ii) recall to measure the ability of a system to present all the relevant names; and (iii) F-measure, which is a harmonic mean of precision and recall.

The next section introduces the NER approaches that are used to identify the mentions of chemical entities in a text accompanied with their bibliographic references of the solutions. The section ends with a table summarising the solution with a consideration of the NER approach used, class of chemical named entity recognised, corpora and performance outcomes.

## Chemical Named Entity Recognition (NER) approaches

**Figure 2:**
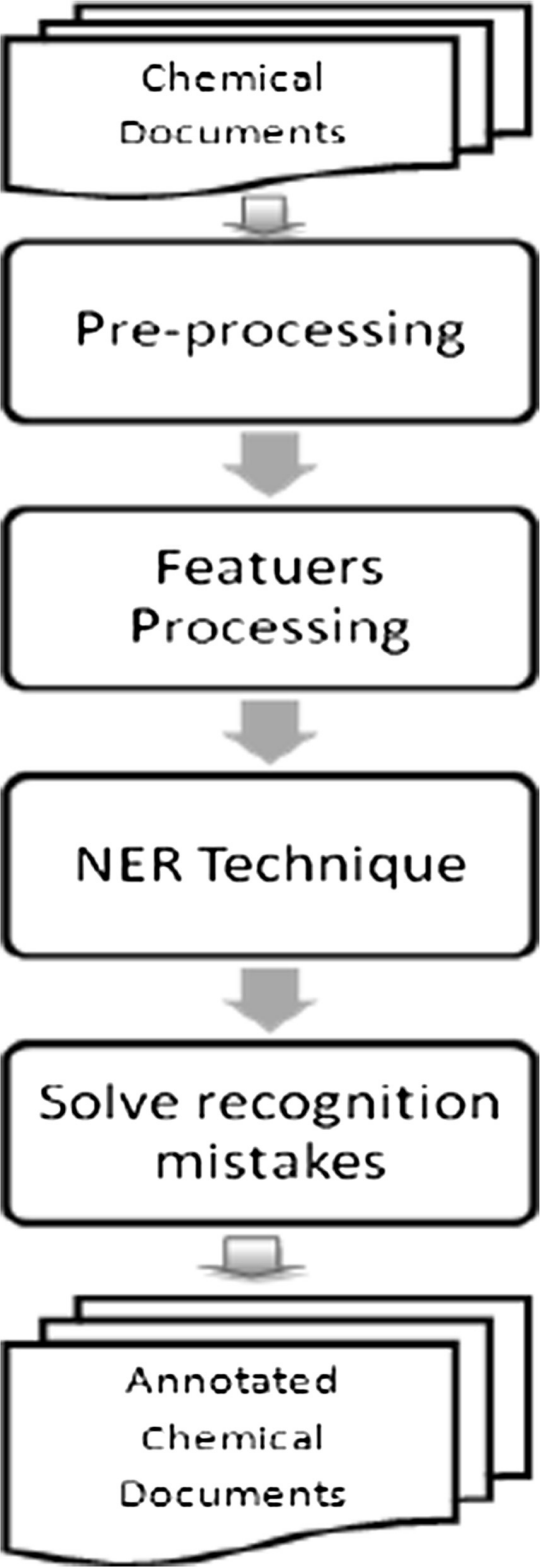
The main steps for developing the chemical NER system.

The term “Named Entity” was introduced in the sixth Message Understanding Conference (MUC6). NER aims to identify the portions of the text that refer to specific entities, such as persons, locations, organisations, etc. It is a subtask of information extraction and the core of the natural language processing (NLP) system [22]. Chemical NER automatically identifies the occurrences of chemical entities in a text. The following steps are required to develop a chemical NER system, and their order is shown in Figure 2.

1. Preprocessing step: This is done to determine entity boundaries in a text by sentence splitting and tokenization.

2. Feature processing step: Due to the complexity of the natural language, creating a set of patterns to match the possible linguistic realizations of the individual facts requires the preprocessing on structural input, such as assigning parts-of-speech and features to words and idiomatic phrases. Table 3 describes the common categories of textual features with some examples.

3. Name recognition step: This recognizes the entity and assigns it to a class or entity type.

4. Solving recognition mistakes or normalization step: This is sometimes addressed as a separate task from the NER. The entity normalization process is represented by mapping entities’ names to their canonical names and by associating them with unique representations so as to help in solving issues resulting from variations in the synonym terms as well as the ambiguous abbreviations [24].

**Table 3:** **Description of common categories of textual features with some examples, summarized from **[23]

**Features categories**	**Objectives and Examples**
Linguistic	to find the prefix that is common to all variations of the term,
	to find the root term of the variant word,
	to assign each token to a grammatical category or
	to divide the text into syntactical correlated parts of words,
	(e.g chucking, lemmatization, stemming and Part-of-speech (POS) tagging)
Orthographic	to capture knowledge on word formation by the presence of these features, (e.g capitalization and symbols)
Morphological	to reflect common structures and/or sub-sequences of characters among entities, (e.g suffixes and prefixes, char n-gram and word shape patterns)
Context	to establish a higher level of relationship between the tokens and the extracted features, e.g (windows and conjunctions)
Lexicons	to add domain knowledge to the set of features for optimizing the NER system. Dictionaries of domain term are used to match the entity names in the text and the resulting tags are used as features. Examples of the types of dictionaries used (target entity name and trigger name).

**Figure 3:**
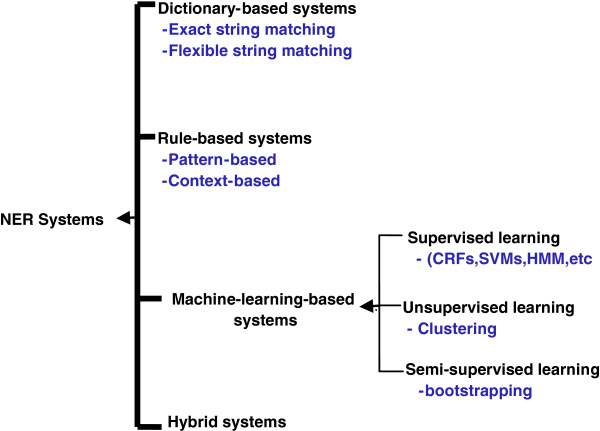
Types of NER systems with some related techniques.

Over the past decades, many automatic NER systems have been developed and used to recognise chemical entities. They are categorised into four groups as shown in Figure 3 and described in the next section.

### Dictionary-based NER systems

A dictionary is a collection of vocabulary for a specific domain usually collected from repositories related to the domain. Dictionaries can be built manually or automatically from public sources, such as databases or thesauri. Examples of dictionaries in the chemistry and biomedicine domains are the Jochem^i^ dictionary [25], which is used to identify small molecules and drugs in the text, and the DrugBank^j^ dictionary for drugs.

Dictionary-based systems use lists of terms in dictionaries to identify the entity occurrences in the text. The system specifies whether a word or a group of words selected from the text matches a term from some dictionary, or implements string-matching algorithms. These algorithms can be divided into two types:

1. Exact matching: This process makes an exact text search for synonyms from a given list of terms against the text.

2. Flexible or approximate matching: This process does not attempt to exactly match the given terms to the text and allows insertion, deletion or substitution for some character(s). It performs fuzzy matching and is used by most NER approaches [8].

The quality of the dictionary-based system depends on the quality and the completeness of the dictionary used as well as the quality of the matching algorithm. Hettne et al. [25] and Rebholz-Schuhmann et al. [26] are examples of dictionary-based systems used to extract drug names and molecules via string matching methods (see Additional file 1). Generally, the dictionary-based method offers high precision but poor recall in cases of spelling errors in the text. This method is further hindered when out-dated dictionaries are changed for the dictionary-based systems because maintaining dictionaries is costly and time-consuming.

### Rule-based NER systems

Rule-based systems [27] use a set of hand-made rules to extract the names of entities. The handcrafted models consist of sets of rules that use grammatical (e.g., parts of speech) and syntactic (e.g., word precedence) rules that are sometimes combined with dictionaries. Two types of rules are usually used in the rule-based systems:

1. Pattern-based rules: These rules depend on the orthographic or morphological patterns of the words.

2. Context-based rules: These rules depend on the context of the words in the text.

An example of a context-based rule is “*If a proper noun follows a person’s title, then the proper noun is a person’s name*” [28]. For example, [29] devised two classes in the chemical NER for identifying biological terms, including two chemical entities. The first class contains chemicals (Indomethacin, N-methylformamide, suberoylanilide and hydroxamic acid); the second class includes the chemical parts, with terms like “methyl groups”, which correspond to the parts of the chemicals. They used pattern-based rules that utilise the orthographic and lexical characteristics of entity classes. For instance, the module used to extract chemical core terms (which have surface features like capital letters, numerals, and special symbols) consists of the recognition of chemical root forms based on IUPAC conventions followed by the chemical naming. For example, consider the sentence “*Polar organic solvents, such as, methanol or N-methylformamide inactivate lipases*.” In this case, *methanol and N-methylformamide* are identified as chemical core terms because they contain the chemical root forms “methyl” and “meth” (refer to Additional file 1 for their results). However, the rule-based NER performs well when the required resources are available (e.g. a set of expert-derived rules), but the systems lack portability. When the data are slightly changed, the high cost of maintaining the rule increases. Some systems can also use dictionaries to improve precision and recall.

### Machine learning (ML)-based NER systems

NER systems that are based on the ML approach [30-32] use statistical models for recognising specific entity names by utilising a feature-based representation of the observed data that depends on the annotated documents. Two basic steps are required to develop the ML-based systems:

1. Training: The machine-learning model must be trained to use the annotations that are present in the annotated documents.

2. Annotating: The documents can be annotated to produce the entity names based on past experience learned from the annotated documents.

However, ML algorithms are categorised based on the desired outcome of the algorithm. The common ML algorithms used un NER are:

#### Supervised learning algorithms

Supervised learning algorithms learn and offer feedback on the learning process (supervised learning) by labelling the training instances with the correct results. For instance, in classification problems that are usually solved by supervised learning, the computer learns the created classification system and produces the output accordingly [33]. For example, the machine-learning–based method for recognising general chemical names in chemical NER proposed in [20] uses Conditional Random Fields (CRFs) [34,35]. A set of five tags (labels) was defined in order to indicate the boundaries of the named entities. These sets were named as follows:

* NO: nonchemical token.

* NE: single-token chemical entity.

* S-NE: start token of a multi-token chemical entity.

* M-NE: middle token of a multi-token chemical entity.

* E-NE: end token of a multi-token chemical entity.

Hence, these labels annotate the training set, and the model has been specifically trained on this set. Thus, the sentence: “. *. . an oligomeric amdioamine salt and an amidoquat …”* in this example would be tagged by the following sequence of tags: NO, S-NE, M-NE, E-NE, NO, NO, and NE.

In chemical NER applications, the supervised learning models, such as CRFs and Hidden Mark Models (HMMs) and Maximum Entropy Markov Models (MEMMs) [36], have received the most research interest in recent years. The next subsections reviews the general characteristics of these models.

HMMs models are characterised by their simplicity, quick learning and the globally made decision of the best sequence after the total analysis of the input sequence [37]. However, when the HMMs are used to label the sequences, they assume the independence of each word from its context, even though this assumption is not true. Thus, HMMs cannot identify the relationships between neighbouring tokens. Another type of supervised models used in the NER are the MEMMs models that take the observation features as inputs and offer better freedom in choosing features to represent observations than the HMM models. However, they suffer from the "label-bias problem”; in this problem, states with low entropy next-state distributions are ignored when observations are made on the conditioning of the data [36].

CRF models differ from the HMMs and MEMMs; they use an undirected graph to avoid the label-bias problem of the MEMMs and ease the conditional independence assumption of the HMMs. Thus, these models have become very popular and are extensively used in many biological and chemical NER applications.

Furthermore, the classification models, such as the Naïve Bayes [6] and the Support Vector Machines (SVMs) [38], make the NER task a classification problem. They are used to classify individual words or multi-word phrases. One of the common tagging schemes is BIO, in which individual tokens are classified (B) as being at the beginning of an entity, (I) being inside the boundaries of an entity, or (O) outside the boundaries of an entity. The main drawback of this scheme appears if the entity boundaries overlap [24]. Many ML-based solutions, such as [2,39-42], are summarised in Additional file 1.

However, supervised models require available inputs, and missing inputs affect the inferring process in the output. Furthermore, the features (such as the textual features described in Table 3) should be extracted and selected. Feature extraction was represented by transforming the text into numerical features applicable for the ML models. Many environments can be used to facilitate the process of feature extraction, such as the frameworks described by [43-45]. The process of selecting a subset of informative and discriminative features to be used on the ML model construction is another very important matter. Feature selection processes affect the performances of algorithms. For example, feature redundancy does not provide the model with more information than the current selected features. Furthermore, using irrelevant features does not provide useful information, whereas the combinations of features may increase performance as in the work of [42]. Usually, after establishing a primary set of features, a set of experiments can be carried out in order to improve the features sets by adding, deleting or modifying features [43].

While most CNER systems used domain-independent feature sets, such as morphological, linguistic, orthographic, context and lexicon features, few studies have examined the impact of these features on the performance effectiveness. Some studies, such as [46] in the newswire domain and [47] in the biomedical domain, have explored the effectiveness of using different features and their combination in NER systems. However, chemical entities differ from newswire entities, particularly in terms of shape features.

Orthographic and morphological features, such as capitalisation, symbols, and word shape patterns, are very important in pattern-based rules and supervised ML CNER approaches because the chemical names contain symbols, roman numbers, dashes, capital and lowercase letters. Furthermore, using orthographical features is advantageous because they provide information to detect the boundaries of the named entities [47].

The windows, capitalisation and dependency parsing features in a supervised CNER system [48] were tested but did not provide positive outcomes. However, the orthographic, morphological and domain knowledge (e.g., dictionary from Jochem) yielded promising results.

Some studies showed that the linguistic features, such as lemmatisers and stemmers, decrease the performance of supervised CNER systems [49], while POS and chuckers are normally used.

Many recent systems have used domain-specific features, such as using additional domain-specific resources (e.g., the drug FDA and ATC nomenclature lists) or outputs of other CNER systems. The results of [49] showed that using domain resource features contributes most to the overall performance.

In addition to the use of these features, tokenisation is an important issue in CNER systems. CNER systems require special types of tokenisers that consider the shapes of chemical entities. For example, the brackets would not be removed from the word “(R)-acetoin”. However, the common tokenisers tokenise the brackets wherever they occur [50].

However, most studies confirmed that using dictionaries and lexicons and token prefix and suffix information features improves the performance of all types of NER systems.

#### Unsupervised learning algorithms

The use of unsupervised learning algorithms seems much more difficult because they aim to teach the computer how to do something without explaining the method, and the labels are not known during training. Thus, the goal of the program in unsupervised learning is to build representations from data. Clustering is an example of unsupervised learning, which aims to find similarities in the training. However, unsupervised learning is not popular in the NER task [22,33].

#### Semi-supervised learning algorithms

Semi-supervised algorithms use both labelled and unlabelled data. These types of systems include a small degree of supervision, i.e., a small set of trusted seeds defined manually for starting the learning process. For example, a system to extract “disease names” is provided with a small number of disease names as relevant examples. The sentences that contain these examples are then searched using the system, which aims to identify contextual clues common to the examples. Other instances appearing in similar contexts are searched again. The learning process is then continually reused for the newly found examples in order to discover new relevant contexts. Thus, a large number of disease names will be recognised by repeating this process [51]. However, to the best of the authors’ knowledge, the unsupervised and semi-supervised learning algorithms have not yet been practically applied in the chemical NER applications.

### Hybrid NER systems

The hybrid NER system implements more than one NER approach in order to utilise the good characteristics from each approach. In the chemical NER, the dictionary approach is usually combined with the rule-based or machine learning approach to improve performance. For example, ChemSpot [52] is a chemical NER tool for identifying mentions of chemical entities (trivial names, drugs, abbreviations, molecular formulae and IUPAC) in text. It implements a hybrid approach that combines a CRF model with a dictionary. The authors stated that the main purpose of the combination was to cover the different naming characteristics of these classes. IUPAC entities are morphologically more complicated than other entities; these entities are difficult to follow in any rule and are best caught by a dictionary. ChemSpot uses the CRF model and the dictionary independently to annotate the text. Finally, the annotations of both approaches are merged. Although the entities extracted by the dictionary or the CRF may overlap, ChemSpot keeps the union of all extracted entities and solves this overlapping by choosing a match from the CRF model. This feature is attributed to the observed higher accuracy of boundary detection by the CRF model. The dictionary component is also used to normalise the extracted entities to the CAS Registry IDs. Another example of the combined system [53] is shown in Additional file 1.

Recent results obtained from the CHEMDNER task of the Fourth BioCreative challenge evaluated the applications of biomedical text mining [21]. CHEMDNER focuses on the recognition of chemical entities (compounds and drugs names) in text. Two subtasks in CHEMDNER are specified in the challenge:

➢ Chemical Document Indexing (CDI): The required output of this subtask is a ranked list of unique chemicals mentioned in a set of given documents.

➢ Chemical Entity Mention (CEM) recognition: The required output of this subtask is the start and end character index pairs for the chemical entities mentioned in a given document.

A text corpus (CHEMDNER Corpus^k^) for training and evaluation purposes was annotated by a domain expert according to particular annotation rules for this task. Approximately 27 teams submitted results. When the obtained automatic annotations were compared against the manual annotation, the best F-scores were 87.39% in the CEM task and 88.20% in the CDI task.

The teams used different technologies in the task, including dictionary lookup in [54], rule-based technologies as in [55,56] and ML methods. The ML algorithm was mostly used with different features, especially CRFs, which were applied by eighteen teams including [48,57-59].

Participants who used lexical resources, such as the Chemical Entities of Biological Interest (ChEBI^l^) ontology, for resolution purposes as in [60] obtained a considerably higher F-score than teams that did not use any lexicon. Furthermore, some systems, such as [49,61], used the outputs of existing systems, e.g., ChemSpot [52] and OSCAR [62], as features in the ML models, which contributed most to the overall system performance leverage. Patterns were also used as features to recognise sequence element symbols (e.g. to cover abbreviations, chemical formulae or chemical identifiers). Semantic information (e.g., UMLS semantic types and ChEBI) were employed via several systems, such as [55], but we observed that the semantic information did not contribute in the performance leverage.

Dictionary-based methods present lower F-scores because they depend on the coverage of the dictionaries. At the same time, creating a dictionary with a high degree of coverage is a difficult matter due to the continuous discovery of novel compounds. The LeadMine system [63] is an example of a hybrid system (dictionary lookup and rule-based). It employed spelling correction, the merging of adjacent entities and entity extension to increase the chance of recognising the trivial names slightly outside the coverage of the dictionary and write rules to describe the systematic chemical nomenclature. The CheNER-BioC [64] is another hybrid system that applied CRFs and dictionary with regular expression taggers to identify formulae and identifier name types. Additional details about the corpus construction, obtained result, technique details and features used on the Biocreative IV challenge can be found in [21].

## Discussion

The examined approaches of NER and their associated applications indicated that each approach features have different requirements and advantages over other approaches. However, dictionary-based systems are more suitable and effective when we have closely defined and updated vocabulary names and when names are correctly written in documents. Otherwise, they can be enhanced by including the potential spellings and orthographic variations or using regular expressions instead of the exact string matching to catch the variability during the matching process [53,65,66]. One of the key advantages of dictionary-based NER approaches is that they allow the normalisation of named entities in one step. When a term is found in the text and disambiguated, it maps directly to the unique identifiers that it represents. In contrast, ML-based NER approaches do not provide identification information of recognised terms [8], which can be solved later by using dictionaries. However, the development and maintenance of comprehensive chemical name dictionaries are nontrivial tasks because an increasing number of new chemicals are being identified as the result of high throughput screening tests and a growing number of other experiments.

Rule-based NER approaches are suitable when the orthographic and morphological structures are strongly defined, but maintaining the rules is costly and time consuming due to the need to cope with the problems of robustness and portability.

In recent years, machine-learning methods have become prevalent to extract chemical entities from the scientific literature. Although machine-learning models rely on the quality of an annotated corpus, they can identify new entities in documents in contrast to the dictionary approaches, which can only identify the entities already present in the recourses. Although ML models are suitable for a variable vocabulary of names, they require large resources. When the appropriate resources are obtainable, the ML approaches perform better than dictionary and rule-based approaches. The ML approaches solve many problems associated with dictionary-based and rule-based approaches by recognising the new entity names, and they perform better in the case of spelling variations in entity names [23]. However, the manual tagging of the training corpus is costly and a non-trivial task, but maintaining the ML-based systems is cheaper than employing rule-based systems.

Using a hybrid NER approach enables us to take advantage of the combined approaches and avoid their associated problems. Hence, the combination of approaches may enhance the targeted performance. Due to the variation in the naming methods of chemical entities, one approach may recognise some types of entities better than other approaches.

Furthermore, a variation in the performance of the summarised solutions in Additional file 1 was observed due the following issues:

➢ Different datasets have been used in the evaluation processes.

➢ Different classes of chemical names (e.g., IUPAC names, trivial names, chemical formula, etc.) are recognised by the different systems.

➢ Some types of chemical names are easier to recognise than others [21], which may result a higher recognition results.

The CHEMDNER task was organised in 2013 (see Chemical Named Entity Recognition (NER) Approaches) due to the previous bottlenecks related to the performance of such systems, such as the difficulty of building a comprehensive dataset with complete annotation guidelines and the heterogeneity of the field and the absence of comparative evaluation efforts for this chemical name recognition task. The variance in the obtained results was deemed suitable and in the boundary of competition.

Biomedical NER applications show a trend towards semi-supervised approaches because they offer more general and independent corpus solutions [23]. Thus, due to a small number of annotated corpora in the chemistry domain for training and testing models, the application of semi-supervised models in chemical NER may enhance the performance because it considers large numbers of un-annotated documents and enables the development of models without relying on training corpora. Hence, applying semi-supervised models for chemical entity recognition may be a focus of future work.

Moreover, most of the work examined the extraction of chemical entities and focused little on its associated data, such as the physicochemical properties and analytical data, which helps to automate or semi-automate the creation of chemical data bases. Other information can be linked to the chemicals, such as biological effects, targets, pharmacokinetic (PK) numerical data and ADME-Tox (absorption, distribution, metabolism, and excretion – toxicity) data. Little work has been performed regarding the extraction of PK and pharmacodynamic (PD) data due to the complexity of the information obtained from the PK/PD studies [67]. In addition to this information, other entities are mentioned in the text with chemical entities, some of which are also chemical but differ in nature, such as genes and proteins or other entities, such as diseases. The extraction of the relationships between these entities is covered in many applications of biomedical text mining, such as the extraction of gene-drug relationships ([68,69], extraction of drug-protein relationships [70], relationship between chemicals and diseases [71] and the relationships between chemical-gene-disease [72].

However, the basic unit of chemical text mining is the recognition of a mentioned chemical entity. Thus, the basis is the development of chemical NER applications characterised with highly effective entity extraction.

## Conclusion

Due to the significant growth of the scientific literature, manually annotating the databases often yields incomplete annotations that are inconsistent with the literature. Developing methods to automatically map text from literature sources to structured forms, such as knowledge bases or databases is an important challenge. In the literature, several techniques are proposed for chemical entity extraction. In this paper, a review of the solutions based on the NER approaches was provided with an outlook on applied approaches and extracted chemical entities. This paper highlighted the types of machine learning models that are not used in chemical NER, such as semi-supervised models, and the information that is not focused upon in the process. The study corroborates existing systems for chemical information extraction that are focused on chemical substances (compounds, reagents, solvents, etc.), but little focus has been given to compound properties and numerical data. Adopting more types of the NER methods, such as, semi-supervised methods, may considerably increase the effectiveness of chemical entity extraction.

## Endnotes

^a^http://www.ncbi.nlm.nih.gov/pubmed

^b^http://www.ncbi.nlm.nih.gov/pmc/

^c^http://www.nlm.nih.gov/

^d^http://www.ncbi.nlm.nih.gov/pccompound

^e^http://www.chemspider.com/

^f^https://scifinder.cas.org

^g^http://www.cas.org

^h^http://www.iupac.org

^i^http://www.biosemantics.org/index.php?page=Jochem

^j^http://www.drugbank.ca

^k^http://www.biocreative.org/tasks/biocreative-iv/chemdner-courpus/

^l^http://www.ebi.ac.uk/chebi/

^m^see Rule-based NER Systems.

^n^this evaluation for chemical names.

^o^OSCAR was evaluated in many corpora by different actors, this evaluation performed by [52].

^p^drugs in this corpus were automatically annotated thus, cannot be considered as gold-standard corpus [52].

## Competing interests

The authors declare that they have no competing interests.

## Authors’ contributions

SE is a PhD candidate and performed the review under the supervision of NS. Both authors read and approved the final manuscript.

## Authors’ information

SE: B. Comp. Sc. (SUST, Sudan), M. Sc. Comp. Sc. (SUST, Sudan), currently Ph. D student at (UTM, Malaysia)

NS: Professor Dr. B. Comp. Sc. (UTM, Malaysia), M. Sc. Comp. Sc. (W. Michigan, US) Ph. D Info. Sc. (Univ. of Sheffield,UK)

## Supplementary Material

Additional file 1 **Summarization for chemical NER solutions starting from 2000 excluding the solutions from BioCreative IV challenge for Chemical NER [**[2,6,7,20,25,26,29,39-42],[50,52,53,62,65,73]. Click here for file
