# Supplementary material for: Chemical named entities recognition: a review on approaches and applications
Source: J Cheminform. 2014 Apr 28;6:17. doi: 10.1186/1758-2946-6-17 (PMC4022577; doi:10.1186/1758-2946-6-17)
Supplement: Additional file 1 — Summarization for chemical NER solutions starting from 2000 excluding the solutions from BioCreative IV challenge for Chemical NER [[2,6,7,20,25,26,29,39-42],[50,52,53,62,65,73]. [file 1758-2946-6-17-S1.doc]

**Additional file 1.** Summarization for chemical NER solutions starting from 2000 excluding the solutions from BioCreative IV challenge for Chemical NER

| Class of NER Approach | Application/Reference | Class of chemical entities extracted | Details of Approach | | | Features Processing | | | | | | | | | | | Evaluation | | | |
| --- | --- | --- | --- | --- | --- | --- | --- | --- | --- | --- | --- | --- | --- | --- | --- | --- | --- | --- | --- | --- |
|  |  |  |  |  |  | POS | Chunking | Normalization | Capitalization | symbols | Suff. & prefix | Char n gram | Word shape | windows | conjunction | lexicons | Corpora | Precision | Recall | F-measure |
| **Dictionary –based Approach** | [25] |  | **Dictionary used** | | **Type of Algorithm** |  |  |  |  | √ | √ |  |  | √ |  |  | SCAI | 51 % | 49% | 50% |
|  |  | IUPAC, partial chemical names, sum formulas, trivial names, abbreviations and chemical family names | Combined information from UMLS, MeSH, ChEBI, DrugBank, KEGG, HMDB and ChemIDplus available at: http://www.biosemantics.org/chemlist | | flexible match |  |  |  |  |  |  |  |  |  |  |  |  |  |  |  |
|  | EBIMed [26] http://www.ebi.ac.uk/Rebholz-srv/ebimed | Drugs and other biological entities | Dictionary compiled from Medline Plus | | flexible match |  |  | √ | √ | √ |  |  |  | √ |  |  | 37 abstracts contains 118 sentence for drug –protein relations | - | - | 74% |
| **Rule-based Approach** | [29] | Two chemical class entities^m^and biological terms | **Rules types** | | | √ |  |  | √ | √ | √ |  |  | √ |  |  | set of corpus selected from MEDLINE abstracts | 93.15% For 1st class | 86.08% For 1st class | 90.86% For 1st class |
|  |  |  | Pattern-based rules | | |  |  |  |  |  |  |  |  |  |  |  |  |  |  |  |
|  |  |  |  |  |  |  |  |  |  |  |  |  |  |  |  |  |  | 100.00% For 2^nd^ class | 84.62% For 2^nd^ class | 91.67% For 2^nd^ class |
|  | ChemFrag [65] | organic chemical names | Pattern-based rules | | |  |  |  |  | √ | √ |  | √ |  |  | √ | patent documents | 91% | 94% | 92% |
| **Machine learning-based Approach** | [39] | Chemical Formula | **Type of Model(s)** | | | √ |  |  | √ | √ | √ | √ |  | √ |  |  | Randomly selected publication | - | - | 58.57% to 92.45% |
|  |  |  | Supervised models(SVM &CRF) | | |  |  |  |  |  |  |  |  |  |  |  |  |  |  |  |
|  | [2] | IUPAC and IUPAC-like names | Supervised model(CRF) | | |  |  | √ | √ | √ | √ |  |  |  | √ |  | SCAI | 86.5% For IUPAC | 84.8% For IUPAC | 85.6% For IUPAC |
|  |  |  |  |  |  |  |  |  |  |  |  |  |  |  |  |  |  | 91.7% For IUPAC-like | 78.6% For IUPAC-like | 84.6% For IUPAC-like |
|  | [6] | Chemical names, analytical data and compound properties | Supervised classification(Naïve Bayes method) | | |  |  |  |  |  |  | √ | √ |  |  | √ | Small corpus selected from Organic and Biomolecular Chemistry | 70.4%^n^ | 78.4% | 74.3% |
|  | [40] | Chemical Formula and names | Supervised model (Hierarchical CRF) | | | √ |  |  | √ | √ | √ |  |  | √ |  |  | Randomly selected publication | 93.09% for formula | 93.88% for formula | 93.48% for formula |
|  |  |  |  |  |  |  |  |  |  |  |  |  |  |  |  |  |  | 76.15% for names | 84.98% for names | 80.32% for names |
|  | [41] | General chemicals | Supervised model(CRF) | | |  |  |  | √ | √ | √ |  |  |  |  |  | 40 manually curated patent documents | 58.5% | 39.5% | 47.2% |
|  | [20] | General chemicals | Supervised model(CRF) | | |  |  |  | √ | √ | √ |  | √ |  |  |  | European Patent Office and the ChEB corpus | 65.76% | 50.35% | 57.03% |
|  | [50] http://www.alias-i.com/lingpipe | Compound, reactions, enzyme, chemical adjective and chemical prefix | Supervised model(HMM) | | |  | √ |  |  | √ | √ | √ | √ |  |  | √ | Full text chemistry papers | 62.7% to 75.3% | 63.4% to 73.5% | 63.1% to 74.4% |
|  | CheNER [42] http://metres.udl.cat/index.php/9-download/4-chener | IUPAC names | Supervised model(CRF) | | |  |  |  | √ | √ | √ | √ | √ | √ | √ |  | SCAI | 82.84% | 77.74% | 80.20% |
|  | [73] | General chemicals | Supervised model( first-order MM) | | |  |  |  |  | √ | √ |  | √ |  |  | √ | set of corpus selected from MEDLINE abstracts | 82.7% | - | - |
|  | Open Source Chemistry Analysis Routines (OSCAR3) [62] http://apidoc.ch.cam.ac.uk/oscar3/ | Compound, reactions, enzyme, chemical adjective and chemical prefix | Supervised model(MEMM) | | | √ | √ |  |  | √ | √ | √ | √ |  |  | √ | SCAI^o^ | 41.4% | 81.6% | 54.9% |
|  | PatentEye [7] | Reactions and spectra data | Supervised model(MEMM) reused from OSCAR3 | | | √ | √ |  |  | √ | √ | √ | √ |  |  | √ | chemical patents from European PatentOffice (EPO) | 78% | 64% | - |
| **Hybrid systems** | DrugNer [36] 53 | Drugs and pharmacologi-cal substances | **Basic Approach Technique** | **Combined Approach Details** | | √ |  |  |  | √ | √ |  | √ |  |  | √ | Drug corpus^p^ | 99.1% | 99.8% | - |
|  |  |  |  | **Dictionary used** | **Type of Algorithm** |  |  |  |  |  |  |  |  |  |  |  |  |  |  |  |
|  |  |  | Pattern-based rules | UMLS Metathesaurus | Exact match |  |  |  |  |  |  |  |  |  |  |  |  |  |  |  |
|  | ChemSpot [52] http://www.informatik.hu-berlin.de/wbi/resources | trivial names, drugs, abbreviations, molecular formulas and IUPAC | (ML) Supervi-sed model (CRF) | ChemIDplus dictionary [25] | flexible match |  |  |  | √ | √ | √ |  |  |  |  | √ | SCAI | 67.3% | 68.9% | 68.1% |
